# Primary central nervous system mucosa-associated lymphoid tissue lymphoma: a diagnostic challenge

**DOI:** 10.1093/bjrcr/uaaf037

**Published:** 2025-07-17

**Authors:** Danial Nasiri, Theoni Maragkou, Andreas Raabe, Anna Katharina Krähenbühl, Franca Wagner

**Affiliations:** Department of Neurosurgery, Inselspital, 3010, Bern, Switzerland; Department of Pathology, Inselspital, 3010, Bern, Switzerland; Department of Neurosurgery, Inselspital, 3010, Bern, Switzerland; Department of Neurosurgery, Inselspital, 3010, Bern, Switzerland; Institute of Diagnostic and Interventional Neuroradiology, Inselspital, 3010, Bern, Switzerland; Department of Otorhinolaryngology, Head and Neck Surgery, Inselspital, 3010, Bern, Switzerland

**Keywords:** primary CNS mucosa-associated lymphoid tissue lymphoma, meningioma, neurosarcoidosis, granulocytic sarcoma/chloroma, dural metastases, Erdheim-Chester disease, hypertrophic pachymeningitis

## Abstract

Primary central nervous system (CNS) mucosa-associated lymphoid tissue (MALT) lymphoma is a rare condition frequently mistaken for meningioma. Since these conditions require distinct treatment approaches, recognizing their imaging characteristics is essential for accurate clinical decision-making. A 69-year-old woman presented with headaches and forehead swelling, prompting MRI of the CNS. Suspecting an intracranial meningioma, the tumour board recommended surgical resection. However, histopathological analysis identified the lesion as a primary CNS MALT lymphoma. Follow-up revealed secondary cutaneous tumour infiltration, leading to a delay in adjuvant radiotherapy. Understanding the differential diagnoses of meningioma is critical for neuroradiologists and neurosurgeons to ensure appropriate treatment planning. This case highlights a misdiagnosis of meningioma that was ultimately identified as a primary CNS MALT lymphoma, emphasizing key imaging and clinical characteristics essential for distinguishing between the most important differential diagnoses of primary CNS MALT lymphoma.

## Introduction

Mucosa-associated lymphoid tissue (MALT) lymphoma is the most common form of extranodal marginal zone B-cell lymphoma and is classified as a non-Hodgkin’s lymphoma.^1^^,^^2^ Primary central nervous system (CNS) MALT lymphoma is a rare condition, accounting for approximately 1% of intracranial tumours.^3^ On imaging, they are mostly misdiagnosed as en-plaque meningiomas due to their similar imaging characteristics on CT and MRI.

Since 1985, more than 100 case reports of primary CNS MALT lymphoma have been documented, in which most lesions were initially presumed to be meningioma, but subsequently identified as primary CNS MALT lymphoma through histopathological analysis.^1^ However, few comprehensive studies have compared primary CNS MALT lymphoma to other conditions with similar presentations, such as neurosarcoidosis, dural metastases, and granulocytic sarcoma/chloroma, or even intracranial manifestations of Erdheim-Chester disease or IgG4-related hypertrophic pachymeningitis. Furthermore, few studies have specifically focused on the differential diagnosis of primary CNS MALT lymphoma based on imaging findings.^4^

Our study aims to clarify the distinguishing features of primary CNS MALT lymphoma on imaging and how to differentiate it from meningiomas and other possible diagnoses. We report a case that was initially mistaken for a meningioma and surgically resected but ultimately characterized as a primary CNS MALT lymphoma upon histological examination. Our goal is to emphasize the importance of recognizing and mentioning rare differential diagnoses in broad-based dural lesions with an extracranial tumour component. This will help to improve therapeutic planning and avoid misdiagnosis on imaging, which could result in inappropriate first-line treatments.

## Case presentation

**Figure 1.**
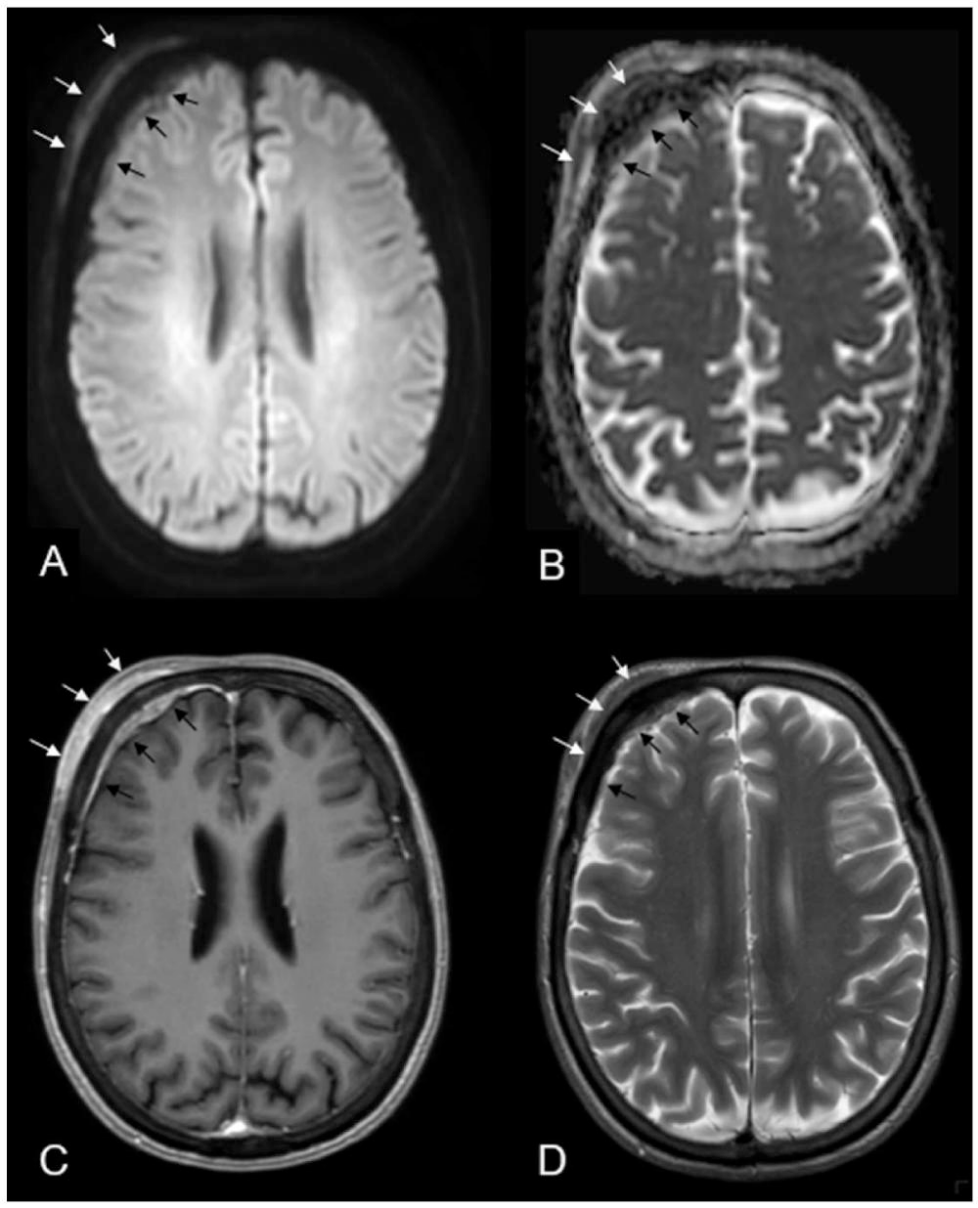
Extra- (white arrows A–D) and intracranial (black arrows A–D) transosseous lesion with diffusion restriction; particularly in the extracranial component, along with very low signal intensity on T2-weighted images and avid, nearly homogeneous contrast enhancement of the extra- and intracranial tumour components.

A 69-year-old woman presented to our outpatient clinic with right-sided forehead swelling and intermittent headaches that had persisted for approximately 6 months. A brain MRI, initially ordered by her primary care physician, revealed a transosseous subgaleal extra- and intracranial en-plaque tumourous lesion. The lesion exhibited diffusion restriction, particularly in the extracranial component, along with very low signal intensity on T2-weighted images and avid, nearly homogeneous contrast enhancement (Figure 1). The adjacent subcutaneous soft tissue on the right forehead appeared unremarkable.

**Figure 2.**
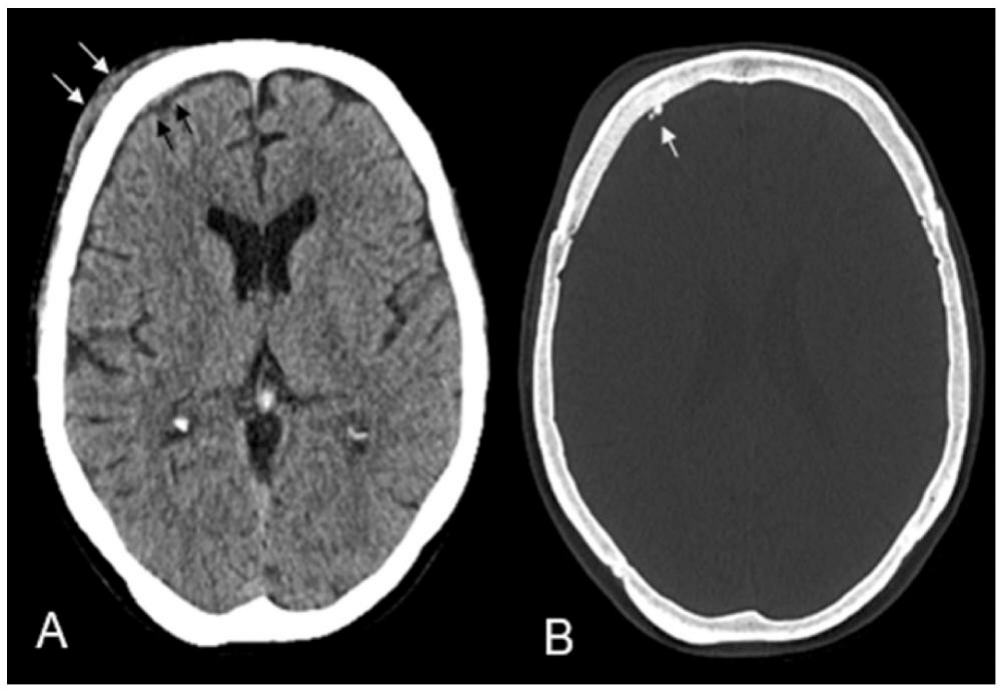
The preoperative CT scan revealed the extracranial subgaleal soft tissue component of the tumour (arrows A) and tiny calcifications along the thickened and enhancing right frontal dura (arrow B). However, the bone marrow of the frontal skull appeared unaffected.

Intracranially, the tumour presented as a narrow extraaxial epidural lamella on the right frontal region with an extended dural tail. At the time of imaging, there was no significant mass effect on the adjacent brain parenchyma or frontal lobe (Figure 1). Given the tumour’s osseous involvement, a CT scan was performed, which revealed tiny calcifications along the thickened and enhancing right frontal dura. However, the bone marrow of the frontal skull appeared unaffected (Figure 2). Based on these imaging characteristics, the leading diagnosis was a transosseous meningioma.

**Figure 3.**
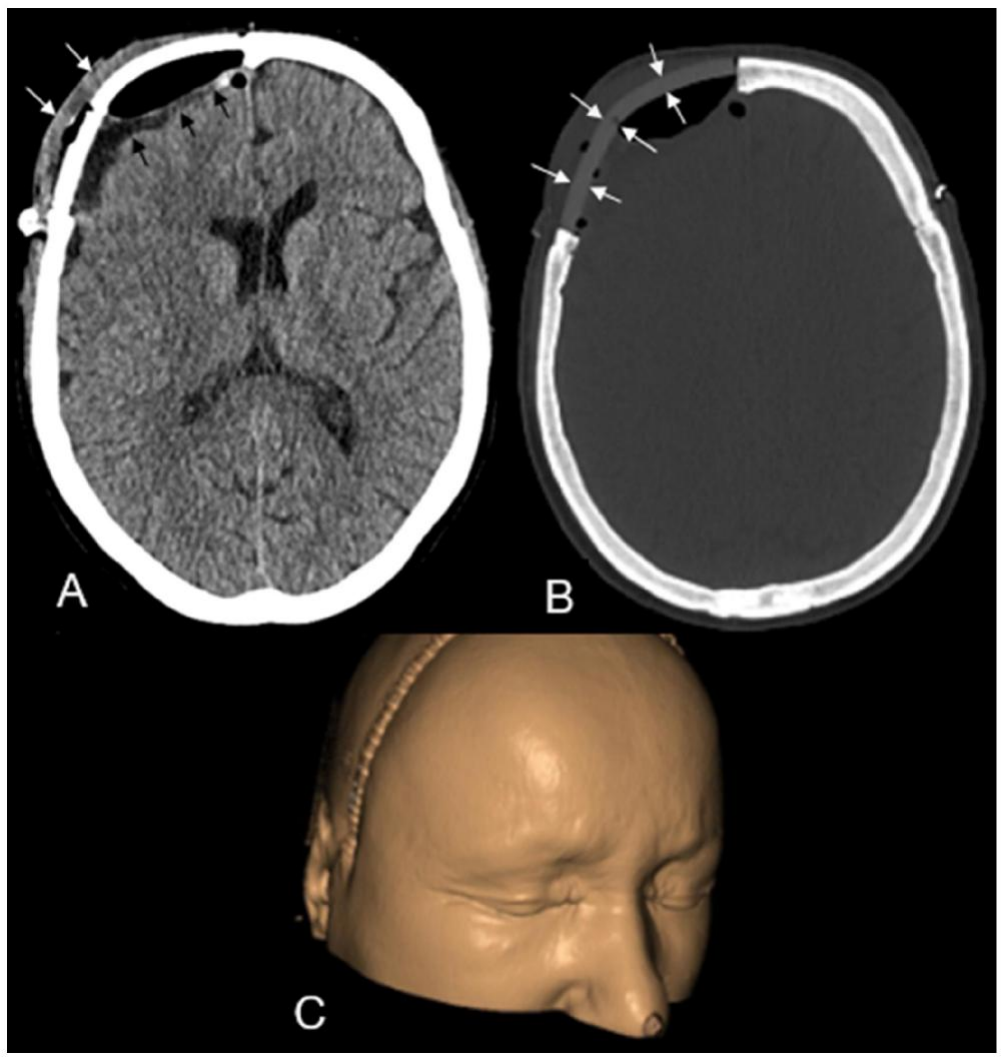
First postoperative computed tomography scan after macroscopic complete resection of the intra- and extracranial tumour components, along with the infiltrated frontal bone, with normal postoperative findings: including intracranial extraaxial gas inclusions and a small extraaxial hygroma with residual lavage fluid (black arrows in A) and mild extracranial soft tissue swelling (arrows in A). B shows the patient-specific implant used to cover the frontal bone defect. Figure 3C shows the 3D-assisted cranioplasty with the skin covered.

Due to the visible forehead swelling, persistent headaches, and radiologic evidence of an intracranial component, surgery was recommended to prevent tumour progression and potential deterioration in the patient’s quality of life. The patient underwent macroscopic complete resection of the intra- and extracranial tumour components, along with the infiltrated frontal bone, with no complications. No new neurological deficits were observed postoperatively. A patient-specific implant was used to cover the frontal bone defect (Figure 3), and a postoperative CT scan confirmed the absence of haemorrhage, residual macroscopic tumour, or other complications (Figure 3).

**Figure 4.**
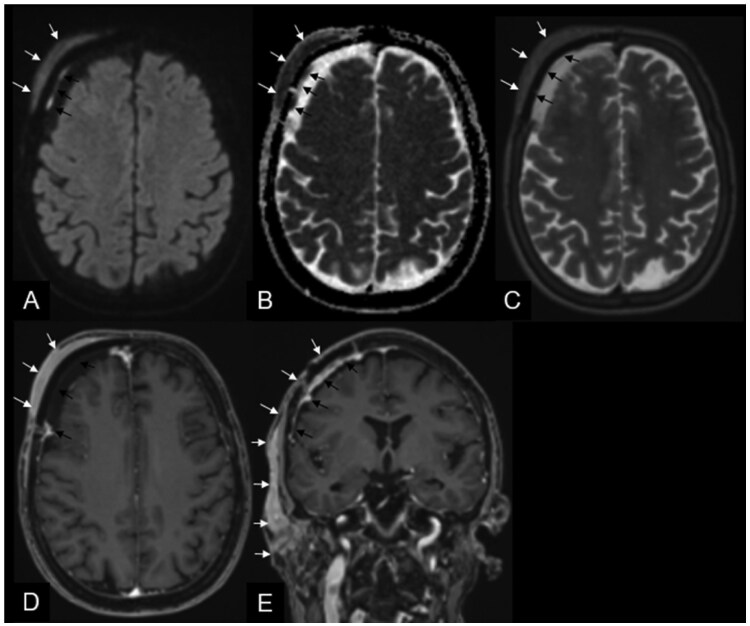
Follow-up magnetic resonance imaging (MRI) 3 months after gross total resection. The brain MRI revealed a soft tissue mass at precisely the same subgaleal location as the primary tumour (white arrows A–E), with new invasion into the adjacent subcutaneous fat and skin. The recurrent tumour had expanded into adjacent anatomical regions, involving the right frontotemporal region, involving the parotid space, directly infiltrating both the superficial and deep lobes of the right parotid gland, and extending to the posterior right masticator space. The MRI characteristics were similar to those of the primary tumour, raising the possibility of intraoperative tumour seeding. The black arrows in A–E point to the patient’s specific implant with primary reactive granulation tissue in the course of the cranioplasty.

Unexpectedly, histopathological analysis identified the lesion as a primary CNS MALT lymphoma. The lymphoma board’s decision was an early follow-up appointment in our outpatient clinic. Three months after gross total resection, the patient returned to the clinic in good neurological condition, without focal deficits but reporting new, progressive soft tissue swelling on the right forehead. An urgent brain MRI revealed a soft tissue mass at precisely the same subgaleal location as the primary tumour, with new invasion into the adjacent subcutaneous fat and skin (Figure 4). The recurrent tumour had expanded into adjacent anatomical regions, involving the right frontotemporal region, the parotid space, directly infiltrating both the superficial and deep lobes of the right parotid gland, and extending to the posterior right masticator space (Figure 4). The MRI characteristics were similar to those of the primary tumour, raising the possibility of intraoperative tumour seeding (Figure 4).

At this stage, a chest and abdominal CT scan was performed to assess systemic tumour spread. No other organ involvement or pathological lymph nodes were detected, suggesting the MALT lymphoma was of primary CNS origin. The multidisciplinary tumour board recommended adjuvant radiotherapy, which was promptly initiated. The patient received a total dose of 24 Gy (12 × 2 Gy) using volumetric modulated arc therapy on a TrueBeam system, targeting the recurrent lymphoma, including the right frontotemporal region and the parotid space. She tolerated the treatment well, without side effects, and showed an immediate response, with visible regression of the forehead swelling and a transition from a purplish-red discolouration to a mild normal hyperemic appearance.

Two months after completing radiotherapy, a fludeoxyglucose-18-positron emission tomography-CT whole-body scan was performed for staging. No metabolically active manifestations of MALT lymphoma were detected, and the previously affected soft tissue areas—including the initial and recurrent tumour sites and the intracranial epidural right frontal compartment—appeared unremarkable.

### Histopathological findings

**Figure 5.**
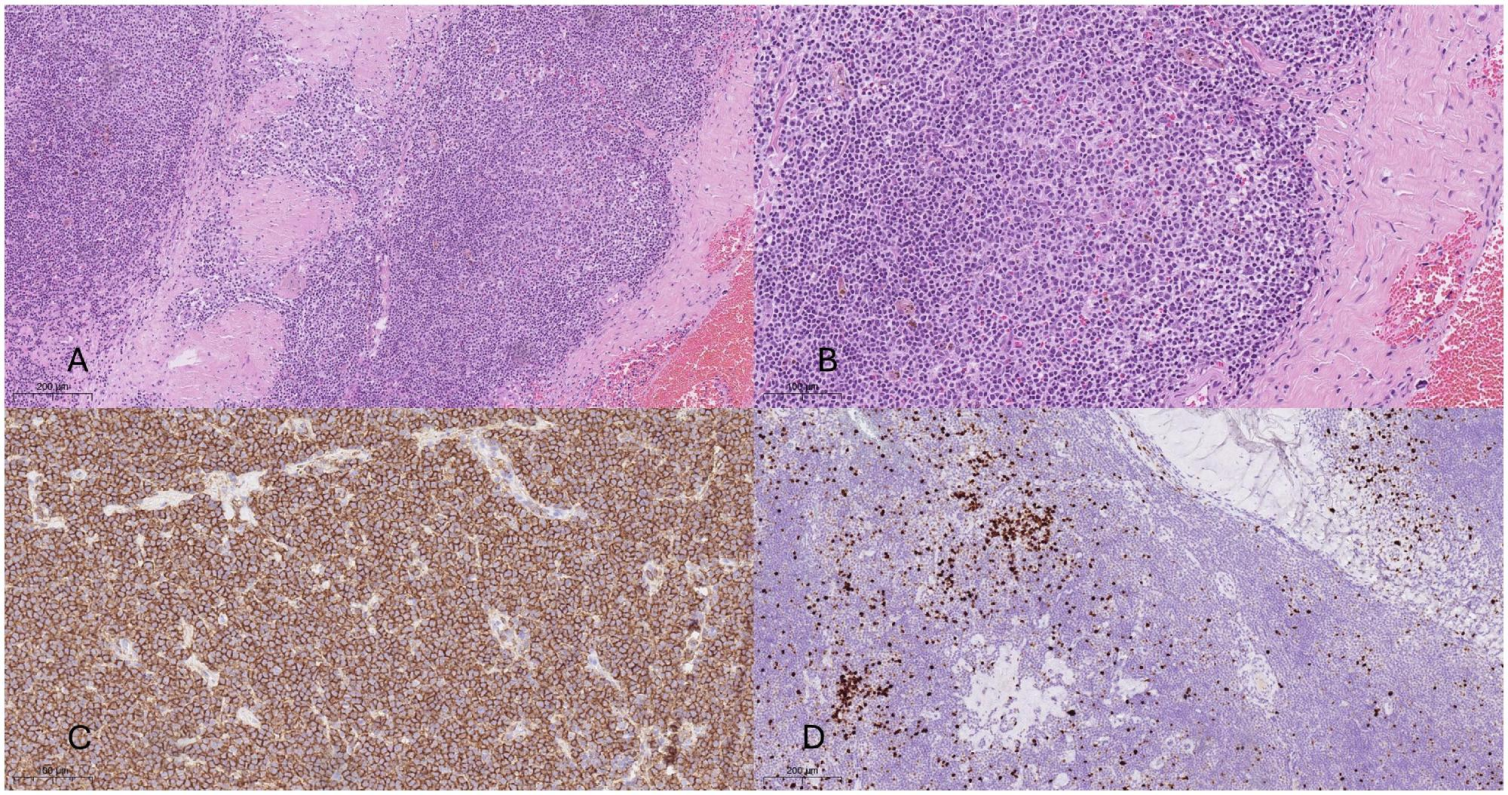
(A and B) Hematoxylin and eosin-stained slices show low- and high-power views of small- to medium-sized lymphoma cells with remnants of residual follicles. (C) The neoplastic B cells reveal an immunohistochemical expression of CD20 antibody. (D) Evaluation of Ki-67 shows a low proliferative activity displayed within the dense lymphoid infiltrates.

The histopathological tissue analysis identified a primary CNS MALT lymphoma composed of small lymphocytes and marginal zone cells, with partial plasmacytic differentiation and residual reactive follicles with follicular colonization. The neoplastic B cells were immunohistochemically positive for CD20, CD19, CD79a, and BCL-2, while CD23, cyclinD1, CD3, CD5, MUM1, BCL-6, CD10, and CD30-tested negative. Ki-67 analysis indicated a low proliferative activity. Figure 5 shows the histopathological analysis.

## Discussion

Our study highlights another case of preoperative imaging misdiagnosis in a rare differential diagnosis, initially suspected to be an en-plaque meningioma but ultimately identified as a primary CNS MALT lymphoma through histopathological analysis following surgical resection. We therefore looked for the most common differential diagnoses for primary CNS MALT lymphoma and their imaging characteristics. A careful comparison of these characteristics can aid neurosurgeons in accurately diagnosing patients presenting with a transosseous extra- and intracranial extraaxial epidural tumour lesion with avid and homogeneous contrast enhancement with a dural tail—the well-known “dural tail” sign.

Most studies claim primary CNS MALT lymphoma to be almost indistinguishable from meningiomas due to their similar appearance with the classical “dural tail” sign, homogeneous and avid contrast enhancement and slow growth.^1^^,^^5^^,^^6^ Furthermore, systemic disorders like IgG4-associated hypertrophic pachymeningitis or Erdheim-Chester disease can mimic the imaging appearance of a primary CNS MALT lymphoma. However, primary CNS MALT lymphoma exhibits some imaging characteristics on MRI that can help in making the correct diagnosis and differentiating them from meningiomas as well as other important differentials such as dural metastases, neurosarcoidosis, and granulocytic sarcoma/chloroma.^4^ Preoperatively, the presence of atypical skull‐bone erosion without bone marrow involvement, T2‐weighted hypointensity on MRI, irregular (non-homogeneous) dural enhancement lacking the classic “dural-tail” sign, or direct invasion of the adjacent brain parenchyma should prompt consideration of primary CNS MALT lymphoma (or other non-meningiomatous lesions) rather than meningioma.

In our case, in line with other reports of primary CNS MALT lymphoma mimicking en-plaque meningioma, the patient was in good overall health with a single-sided tumour manifestation. She had a few age-related comorbidities but no signs of systemic cancer or inflammatory disease, making dural metastases, a granulocytic sarcoma/chloroma, or systemic disorders seem unlikely.^7^ Understanding the overall clinical context is the most crucial first step in arriving at the correct diagnosis. Differential diagnosis for the major differentials mentioned, especially meningioma, can significantly alter treatment approaches. Table 1 gives an overview of the typical imaging and clinical characteristics of primary CNS MALT lymphoma in comparison to the most important differential diagnoses.^8–13^

**Table 1.** Imaging and clinical characteristics of differential diagnoses for CNS MALT lymphoma.

	MRI DWI	MRI T1w	MRI T2w	MRI T1w C+	CT bone	Clinical signs and hints
Primary CNS MALT lymphoma	Strong DWI-restriction	Isointense to cortex	Iso- to hypointense to cortex; surrounding parenchymal/vasogenic oedema in most cases	Avid and strong, homogeneous enhancement	Hyperdense compared to the cortex	Indolent tumour, usually clinical signs such as headache, focal-neurological deficits, cranial nerve palsy or seizures related to mass effect and oedema
Meningioma	Possible slight DWI- restriction in benign entities, strong diffusion restriction in anaplastic/malign subtypes	Isointense to cortex	Usually, iso-to-mild hyperintense to cortex	Homogeneous strong enhancement, “dural tail” sign	Calcifications, focal hyperostosis, or bony arrosion	Seizures, cranial nerve palsies, headache and/or changes in mental status
Neurosarcoidosis	High signal intensity in the acute inflammatory stage and DWI may demonstrate recent ischemia	Iso-/hypointense to cortex	Variable, most hyperintense to cortex	Multifocal avid enhancing dural lesions, leptomeningeal enhancement, hypothalamus and pituitary involvement and/or cranial nerve involvement	Usually unremarkable, rare “punched out” lesions of the skull	Systemic disorder: seizures, focal neurological deficits, cranial nerve palsies, endocrine dysfunction (eg, diabetes insipidus; amenorrhea)
Granulocytic sarcoma/chloroma	Strong DWI restriction	Iso-/hypointense to cortex	Iso-/mildly hyperintense to cortex	Intensive homogeneous enhancement with potential parenchymal invasion	Lytic sharply delineated lesion	Primary chloroma is very rare, usually occurring in patients with myeloproliferative and myelodysplastic disorders, most acute myeloid leukaemia
Dural metastasis	Restricted diffusion	Iso-/hypointense to cortex	Iso-/hyperintense to cortex	Multifocal nodular dural vivid enhancement and dural thickening	May show calvarial invasion depending on the primary malignancy	Dural metastases are typically multiple; clinically, patients present with headache, fatigue, confusion, and focal neurology
Erdheim-Chester disease	No restriction	Iso- to mildly hyperintense to cortex	Hypointense to cortex	Intense contrast enhancement	Facial or skull bone thickening, maxillary, sphenoid sinus wall or ethmoidal cell wall osteosclerosis	Hypothalamic-pituitary and orbital involvement common, symptoms depending on the intracranial findings (eg, diabetes insipidus common)
Hypertrophic pachymeningitis	Mild DWI-restriction	Hypointense to cortex	Hypointense to cortex	Dural thickening might be focal or diffuse with avid homogeneous enhancement	Plaque-like/focal nodular dural thickening	IgG4-related hypertrophic pachymeningitis is becoming increasingly recognized, secondary to various aetiologies, including rheumatological diseases; most commonly presenting with multiple recurrent cranial neuropathies and daily headache

Abbreviations: CT = computed tomography/bone window; DWI = diffusion-weighted imaging; MRI = magnetic resonance imaging; T1w = T1-weighted imaging; T2w = T2-weighted imaging; T1w C+ = T1-weighted imaging after contrast application.

In our case, surgical resection of the presumed en-plaque meningioma was recommended, and the procedure proceeded without complications. During its postoperative discussion, our lymphoma board recommended an early follow-up check-up in our outpatient clinic. However, by that time, the patient had already developed a subcutaneous recurrence of her primary CNS MALT lymphoma, presumed to result from intraoperative tumour seeding with involvement of the parotid space. Given that the patient’s only symptoms at initial presentation were a right-sided forehead swelling and recurrent headaches, primary radiotherapy, rather than surgery, might have been more appropriate after a minimally invasive biopsy for histopathological assessment of the entity.^14^ Moreover, open surgery with total gross tumour resection carries the risk of intra- and postoperative complications as well as the potential for cancer cells to spread to the skin or adjacent bone, leading to tumour relapse.^14^

Many case reports suggest that surgery is one option for treating primary CNS MALT lymphoma, the others being radiotherapy and chemotherapy.^15^ This is particularly interesting because treatment for localized CNS lymphoma typically involves non-surgical approaches, with involved site radiotherapy (ISRT) or ISRT plus whole-brain radiotherapy (WBRT) being first-line treatments.^2^ Rituximab, chemotherapy, or combined chemoimmunotherapy alongside radiotherapy is typically reserved for cases with multifocal disease or within “watch-and-wait” strategies, where only symptomatic lesions are targeted.^2^

However, even when a dural‐based lesion presents with classic meningioma features—well-circumscribed margins, a dural tail, homogeneous enhancement, and minimal brain invasion—non-invasive imaging cannot reliably distinguish MALT lymphoma from meningioma in every case. Subtle differences in diffusion characteristics or perfusion metrics may hint at lymphoma, but overlap is common, and stereotactic biopsy can be technically challenging or inconclusive for superficial, broad-based lesions. In practice, when a dural mass is easily accessible, causing significant mass effect or edema, and radiographic assessments remain equivocal, neurosurgical resection serves a dual purpose: it secures sufficient tissue for an accurate diagnosis and provides immediate cytoreduction. Gross total resection of dural MALT lymphoma, followed by focal low-dose radiotherapy and, where appropriate, CD20-directed immunotherapy, has demonstrated excellent local control and durable remission, justifying this pragmatic, combined-modality approach in lesions masquerading as meningiomas.

Our case emphasizes the invasiveness of surgical resection, especially when the skull is involved. We replaced the patient’s bone with a patient-specific implant to ensure complete tumour removal. For asymptomatic or oligosymptomatic patients, a thorough systemic check-up should be conducted to assess the differential diagnoses mentioned above. Such considerations can significantly alter the course of therapy and impact recommendations made by neurooncological tumour boards.

## Conclusion

Primary CNS MALT lymphoma is a rare condition frequently misdiagnosed as en-plaque meningioma, its primary differential diagnosis, due to overlapping imaging features. This radiological misinterpretation can lead to surgery being prioritized as the initial treatment approach. Neuroradiologists and neurosurgeons should be well-versed in the distinct imaging characteristics of primary CNS MALT lymphoma and those of its differentials, including meningioma, dural metastases, neurosarcoidosis, granulocytic sarcoma/chloroma, and systemic disorders such as Erdheim-Chester disease or hypertrophic pachymeningitis. Imaging findings must always be assessed within the clinical context, considering patient history, to prevent misdiagnosis.

## Learning points

- Primary CNS MALT lymphoma may resemble meningioma in imaging diagnostics.
- Neuroradiologists and neurosurgeons should be aware of rare but clinically and therapeutically significant differential diagnoses of meningioma to ensure optimal treatment selection.
- Given the distinct treatment approaches for primary CNS MALT lymphoma and meningioma, misclassification may lead to inadequate therapy. In surgically treated cases, local recurrence of lymphoma could potentially be attributed to tumour seeding.
- A multidisciplinary board discussion is crucial for making optimal treatment decisions both before surgery and after surgical resection.
